# The correlation analysis between sagittal alignment and cross-sectional area of paraspinal muscle in patients with lumbar spinal stenosis and degenerative spondylolisthesis

**DOI:** 10.1186/s12891-019-2733-7

**Published:** 2019-07-31

**Authors:** Akihiko Hiyama, Hiroyuki Katoh, Daisuke Sakai, Masahiro Tanaka, Masato Sato, Masahiko Watanabe

**Affiliations:** Department of Orthopaedic Surgery, Tokai University School of Medicine, 143 Shimokasuya, Isehara, Kanagawa 259-1193 Japan

**Keywords:** Skeletal muscle mass (SMM), Cross-sectional area (CSA), Spinal alignment, Pelvic tilt (PT), Bioimpedance analysis (BIA)

## Abstract

**Background:**

The relationship between spinal alignment and skeletal muscle mass (SMM) has attracted attention in recent years. Sagittal alignment is known to deteriorate with age, but it is not known whether this is related to paraspinal muscles. Therefore, the purpose of this study is to elucidate the role of the multifidus (MF) and psoas major (PS) muscles in maintaining global spinal alignment in patients with lumbar spinal stenosis (LSS) and/or degenerative spondylolisthesis (DS), and to analyze whether each muscles’ cross-sectional area (CSA) correlates with whole-body SMM using bioimpedance analysis (BIA).

**Methods:**

We retrospectively evaluated 140 patients who were hospitalized for surgery to treat LSS and/or DS. Spinal alignment, CSA of spinal muscles, and body composition parameters were measured from full-length standing whole-spine radiography, MRI, and BIA before surgery. The following standard measurements were obtained from radiographs: sagittal balance (C7-SVA), cervical lordosis (CL; C2–C7), lumbar lordosis (LL; L1–S1), thoracic kyphosis (TK; T5–T12), pelvic incidence (PI), pelvic tilt (PT), and sacral slope (SS).

**Results:**

The average PS CSA (AveCSA) was highest at L4-L5, whereas MF AveCSA was highest at L5-S1. Paraspinal muscle CSAs were greater in males than in females. There was no statistically significant difference between the left and right CSA for either MF or PS. Correlation coefficient showed strong correlations between the PS AveCSA (L4-L5) and whole body SMM (r = 0.739). Correlation coefficient analysis also showed weak correlation between SMM and PT (r = − 0.184). Furthermore, PS AveCSA (L4-L5) correlated with the PT (r = − 0.183) and age (r = − 0.156), while PT correlated with the whole body SMM (r = − 0.184) but not with age.

**Conclusions:**

Whole body SMM showed correlation with PS AvCSA (L4-L5) and with PT among the spinal parameters, which was the same result in MF AvCSA (L4-L5). These findings suggest that the posterior inclination of the pelvis may be correlated with paraspinal muscle area rather than age.

## Background

Muscle mass decreases with age. Observational studies have shown an annual decline of approximately 1% after the age of 40 [1]. In recent years, the relationship between low back pain (LBP) and the mass of trunk muscles, including skeletal muscles, has attracted attention. The density of the paraspinal muscles and their cross sectional area (CSA) size are known to be associated with variables such as age, gender, and weight [2–4]. It is generally agreed that muscle CSA and density reflect muscle performance and physical function of individuals [5].

Various imaging techniques (computed tomography, magnetic resonance imaging (MRI), and ultrasound) have been reported as reliable and useful tools for measuring CSA, density, and fat infiltration of paraspinal muscles [3, 6–8]. Bioimpedance analysis (BIA) has been used in various contexts for the measurement of the nutritional components of body composition, such as fat mass or fat-free mass, using the electrical properties of body tissues. It is easy, non-invasive, relatively inexpensive, and can be performed in almost any subject because of its portability [9]. It has also recently shown promise as a tool for the measurement of water volume status of body [10, 11].

The alteration in sagittal alignment is thought to be one of the potential risk factors influencing disorders of the lower back. Especially in older people, spinal sagittal malalignment causes poor health-related quality of life (QOL) [12].Various factors affecting spinal alignment have been studied, including patient demographics (gender and age) and radiographic factors [13, 14]. Generally, sagittal imbalance results in increased muscular effort and energy expenditure, causing pain, fatigue, and disability. Yagi et al. reported that trunk muscles play an important role in spinal structure and, based on the evaluation of the CSA in spinal deformity, that paraspinal muscle degeneration is related to spinal deformity [15]. However, the relationship between body composition measured by BIA and paraspinal muscle CSA measured by MRI in patients with spinal disease has not been studied, and their correlation with spinal alignment is not clear. Therefore, this study was conducted to elucidate the role of the multifidus (MF) and psoas major (PS) muscles in maintaining global spinal alignment in patients with lumbar spinal stenosis (LSS) and degenerative spondylolisthesis (DS), and to analyze whether muscle CSA correlates with SMM measured by BIA.

## Methods

### Included patients

We retrospectively evaluated patients who were hospitalized for surgery to treat lumbar spinal stenosis (LSS) and degenerative spondylolisthesis (DS) from October 2015 to April 2019. The study group included 288 patients (171 male and 117 female) who were diagnosed with LSS and/or DS and were evaluated by BIA [16], MRI, and whole-spine posteroanterior and lateral full-spine radiographs. Five spinal surgeons diagnosed degenerative spine disease based on subjective symptoms, neurological findings, and MRI. Adult spinal deformity patients with coronal Cobb angles above 30 degrees were excluded.

**Fig. 1:**
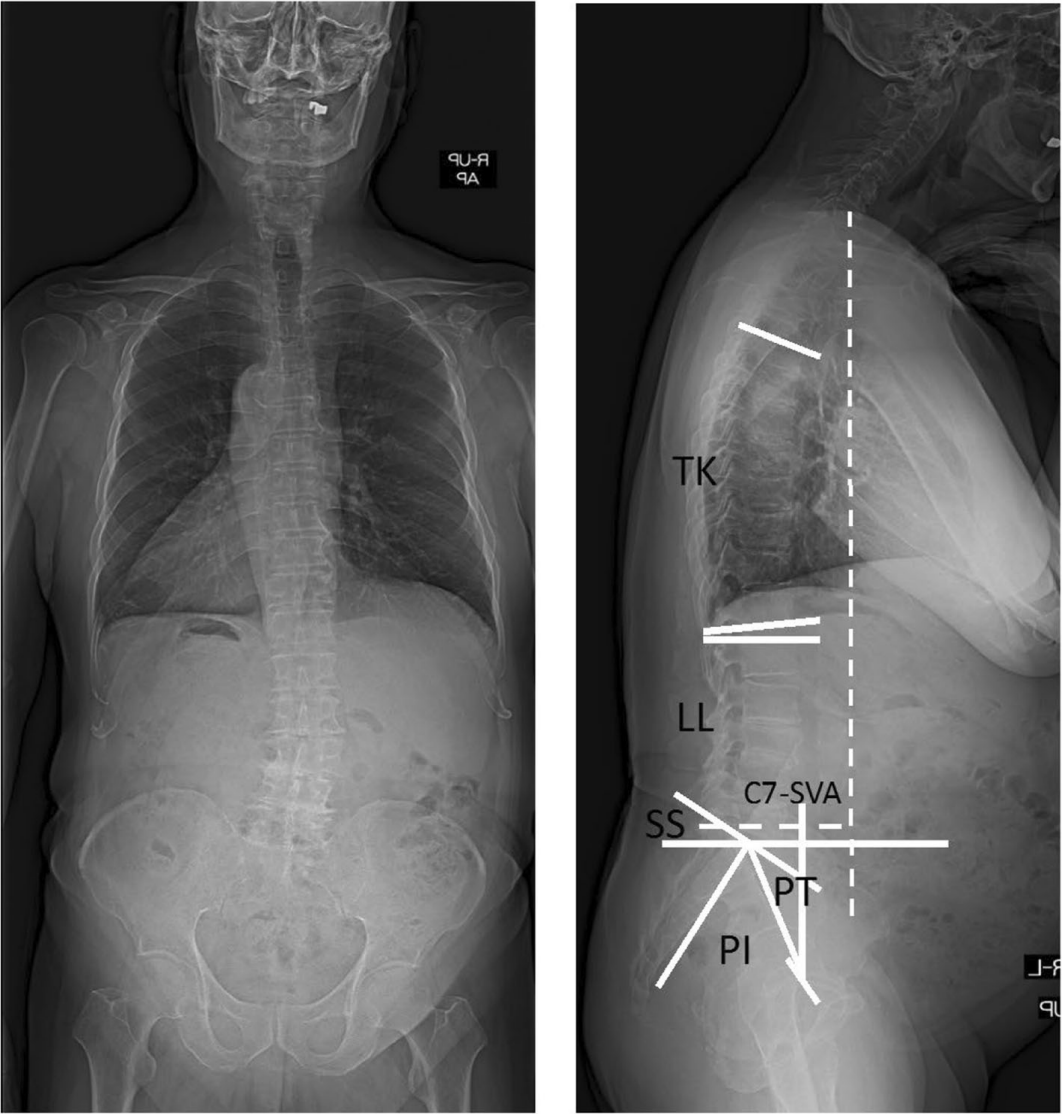
Standing-erect whole-spine posteroanterior and lateral full-spine radiographs. Measurement of sagittal parameters. Thoracic kyphosis (TK) was the Cobb’s angle between upper end plate of T5 and lower end plate of T12. Lumbar lordosis (LL) was the Cobb’s angle between upper end plate of L1 and S1. Pelvic incidence (PI) was the angle between the perpendicular to the sacral plate at its midpoint and the line connecting this point to the middle axis of both femoral heads. Pelvic tilt (PT) was the angle between the line connecting the midpoint of the sacral plate to the axis of the femoral heads and the line perpendicular to the floor. Sacral slope (SS) is defined as the angle subtended by the horizontal line and upper sacral end plate. Sagittal vertical axis (C7-SVA) was the distance between the C7 plumb line and the postero- superior corner of S1

X-ray evaluations involved examination of standing whole-spine posteroanterior and lateral full-spine radiographs. For the lateral films, the patients stood with their knees locked and fully extended when possible, the feet shoulder-width apart, looking straight ahead, and with their elbows bent and knuckles in the supraclavicular fossa bilaterally (Fig. 1). Body composition was measured using Inbody S20 (Biospace Inc., Seoul, Korea), which is a bedside body composition analyzer for patients who cannot stand.

Clinical outcomes, radiological parameters, body composition analyses, and patient characteristics including sex, age, height, body weight, body mass index (BMI), and a numeric rating scale (NRS) of LBP were examined. Analysis of the correlation between skeletal muscle mass (SMM) of the whole body and each sagittal parameter was performed for all patients.

### Radiological parameters

Radiographic parameters of interest included sagittal balance (C7-SVA), cervical lordosis (CL; C2–C7), lumbar lordosis (LL; L1–S1), thoracic kyphosis (TK; T5–T12), pelvic incidence (PI), pelvic tilt (PT), and sacral slope (SS) [17, 18]. The C7-SVA was determined by the horizontal offset between a plumb line drawn from the center of C7 and the posterosuperior corner of the S1 endplate. The CL was measured as the angle between the lower endplates of C2 and C7. The LL was the sagittal cobb angle measured between the superior end plate of L1 and the superior end plate of S1. The TK was measured from the upper endplate of T5 to the lower endplate of T12. The PI was measured as the angle between a line drawn perpendicular to the sacral end plate at its midpoint and a line drawn from the midpoint of the sacral end plate to the midpoint of the femoral head axis. The PT was measured as the angle subtended by the lines drawn from the center of bicoxofemoral axis to the mid-point of sacral endplate and a vertical line drawn from this point [19].

### Bioelectric impedance analysis

BIA is a commonly used method for estimating body composition, in particular body fat and muscle mass [9]. The Inbody S20 analyzer measures the electrical responses at multiple frequencies between 1 and 1000 kHz and estimates extracellular water (ECW) and total body water (TBW) in accordance with reactance and resistance by the method described by Chamney et al. [10]. The measurements were performed with the patient in the supine position using eight hand and foot tactile electrodes. The input variables included the patients’ age, sex, height, and actual body weight. The volume status was expressed as ECW/TBW.

### Evaluation of muscles in MRI

**Fig. 2:**
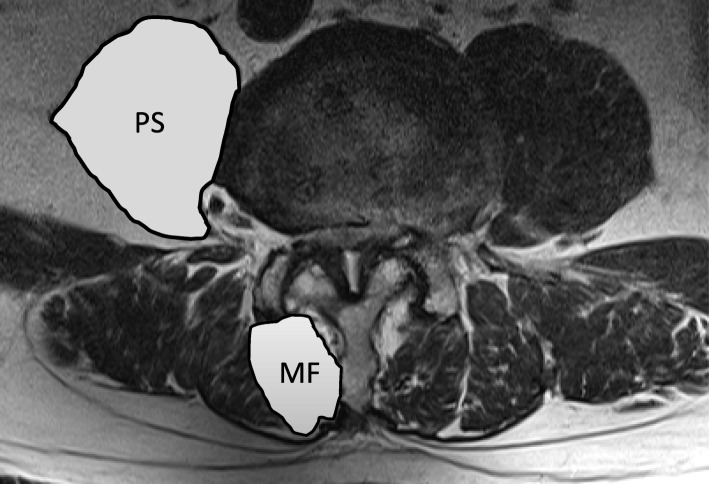
Axial slice by spinal levels. Measurement of cross-sectional area (CSA) of psoas (PS) and multifidus (MF)

We used a 1.5 or 3.0-T imaging system (Ingenia or Achieva; Philips Medical Systems, Best, the Netherlands) for MRIs in this study, Sagittal images were taken for the entire spine, but axial images were for each lumbar intervertebral level parallel to the vertebral endplates. Three preoperative T2-weighted axial images from the L3–4, L4–5, and L5–S1 intervertebral disc levels were used to measure PS and MF CSA and analyze muscle size and morphology. The bilateral CSAs of the MF and PS muscles at each intervertebral disc level were determined by outlining the fascial boundary of the muscles and using the measurement function of the image processing software. All images were stored in DICOM file format on a Picture Archiving and Communication System (PACS) (Fig. 2).

### Statistical analysis

Statistical analysis was performed using IBM SPSS Statistics version 20.0 (IBM Corp., Armonk, NY). All values are expressed as mean ± standard deviation. An analysis of variance with a posthoc test (Mann–Whitney *U* test) was used for comparisons. The correlations between SMM and sagittal alignment were analyzed using Spearman’s product-moment correlation coefficient.

Intraobserver reliability and the interobserver reliability were evaluated using the intraclass correlation coefficient (ICC) [20]. The results between > 0.8 were defined as excellent, between 0.6 and 0.8 as good, between 0.4 and 0.6 as moderate and < 0.4 as bad correlation between two values.

A power analysis was performed using the G-Power Analysis software program (G Power 3.1.9, University of Düsseldorf, Germany, http://www.gpower.hhu.de/en.html) [21] to calculate the minimum sample size necessary to detect a difference between two independent groups (calculated with Cohen’s d = 0.55, alpha = 0.05, two-tailed, power = 0.80) indicated a required sample size of 42 participants. A power analysis performed to calculate the minimum sample size necessary to detect a correlation (calculated with effect size = 0.3, alpha = 0.05, power = 0.80) indicated a required total sample size of 82 participants. For all statistical analyses, the type 1 error was set at 5% and *p* < 0.05 was considered significant.

## Results

Of the included 288 patients, complete analysis was possible in 140 patients. Patients were excluded when MRI images were insufficient to measure CSAs of the MF and PS muscles or when spinal parameters could not be accurately measured on radiographs. The field of view in axial images were unfortunately often too narrow to measure PS CSA especially at the L5-S1 level, leading to exclusion of many cases.

The demographics and radiological parameters of the 140 patients are listed in Table 1. The mean age at the time of operation was 70.6 ± 9.0 years and 52.1% of the patients were female. Height and body weight were significantly higher in males compared to females, while the spinal parameters PI and PT were significantly higher in females compared to males.

**Table 1:** Summary of characteristics in 140 study patients. BMI; body mass index, NRS; numeric rating scale, LBP; low back pain, CL;cervical lordosis (C2–C7), TK; thoracic kyphosis (T5–12), LL; lumbar lordosis (T12–S1), SS; sacral slope, PI; pelvic incidence, PT; pelvic tilt, SS; sacral slope, SVA; sagittal vertical axis. All values are expressed as mean value ± standard deviation

	Overall	Male	Female	*P* value
Number of Cases	140	67	73	
Age (yrs)	70.6 ± 9.0	70.9 ± 9.4	70.4 ± 8.6	0.724
Height (cm)	158.2 ± 9.6	165.0 ± 7.9	152.0 ± 6.3	< 0.001
Body weight (kg)	61.5 ± 12.8	67.5 ± 12.8	56.1 ± 10.3	< 0.001
BMI (kg/m^2^)	24.4 ± 4.0	24.7 ± 3.3	24.2 ± 4.5	0.153
NRS of LBP	5.5 ± 2.8	5.6 ± 2.8	5.5 ± 2.7	0.984
Radiological parameters				
CL	8.9 ± 14.3	9.4 ± 15.1	8.4 ± 13.8	0.358
TK	25.2 ± 9.8	25.7 ± 9.1	24.8 ± 10.4	0.809
LL	32.2 ± 15.8	31.7 ± 14.4	32.7 ± 17.0	0.404
PI	50.1 ± 9.6	47.8 ± 8.6	52.2 ± 10.1	< 0.01
PT	23.6 ± 9.0	21.5 ± 7.8	25.5 ± 9.6	< 0.05
SS	26.0 ± 9.2	25.9 ± 10.0	26.2 ± 8.5	0.470
C7-SVA	65.4 ± 55.1	67.6 ± 51.7	63.4 ± 58.4	0.490

BIA measurements are shown in Table 2. The SMM, soft lean mass, protein and mineral measured by BIA were significantly higher in males compared to females. ICW and ECW also had similar results.

**Table 2:** Results of BIA body compositions analysis, muscle-fat analysis, obesity estimation, and body water analysis. SMM; skeletal muscle mass, BIA; bioimpedance analysis, ICW; intracellular water, ECW; extracellular water, TBW; total body water. All values are expressed as mean value ± standard deviation

BIA	Overall	Male	Female	*P* value
ICW (ℓ)	19.1 ± 4.4	22.1 ± 3.8	16.4 ± 3.0	< 0.001
ECW (ℓ)	12.4 ± 2.7	14.2 ± 2.2	10.8 ± 1.9	< 0.001
ECW/TBW (Leg)	0.398 ± 0.015	0.396 ± 0.015	0.399 ± 0.014	0.157
ECW/TBW (Whole body)	0.395 ± 0.012	0.393 ± 0.012	0.397 ± 0.011	< 0.05
Protein (kg)	8.3 ± 1.9	9.5 ± 1.7	7.1 ± 1.3	< 0.001
Mineral (kg)	2.9 ± 0.6	3.3 ± 0.6	2.6 ± 0.4	< 0.001
Soft lean mass (kg)	40.3 ± 9.1	46.4 ± 7.8	34.6 ± 6.2	< 0.001
Skeletal muscle mass (SMM) (kg)	22.9 ± 5.8	26.8 ± 5.0	19.4 ± 3.9	< 0.001
Body fat mass (kg)	18.7 ± 7.8	18.4 ± 8.0	19.0 ± 7.7	0.422
Percent body fat (%)	30.1 ± 9.3	26.6 ± 7.9	33.3 ± 9.3	< 0.001

CSA measured by MRI revealed no difference found between the right and left sides, but demonstrated that CSA of both PS and MF muscles were significantly higher in males compared to females. PS AveCSA was highest at the L4-L5 level, whereas MF AveCSA was the highest at the L5-S1 level (PS AveCSA; L3-L4, 770.8 ± 304.6 mm^2^; L4-L5, 1005.8 ± 333.9 mm^2^; and L5-S1, 887.1 ± 319.5 mm^2^. MF AveCSA; L3-L4, 358.9 ± 106.6 mm^2^; L4-L5, 464.9 ± 140.5 mm^2^; and L5-S1, 484.1 ± 144.6 mm^2^) (Table 3). Measurement of the CSA of both PS and MF muscles using MRI showed to excellent intraobserver reliability (PS AveCSA; L3-L4, 0.905; L4-L5, 0.945; and L5-S1, 0.981. MF AveCSA; L3-L4, 0.914; L4-L5, 0.905; and L5-S1, 0.919)).

**Table 3:** Comparison of PS and MF CSAs by spinal levels. PS, Psoas; MF, Multifidus; CSA, Cross-sectional area. All values are expressed a mean ± standard deviation. *** *p*< 0.001 indicates significant differences between groups

PS (mm^2^)					*P*	MF (mm^2^)			*P*
		Overall	Male	Female		Overall	Male	Female	
	L3-L4	751.6 ± 300.5	964.3 ± 251.7	556.4 ± 188.4	***	351.3 ± 113.1	402.6 ± 97.0	304.2 ± 106.7	***
Left	L4-L5	1001.3 ± 329.8	1237.0 ± 289.0	784.8 ± 185.1	***	459.3 ± 148.6	542.3 ± 135.5	382.2 ± 115.5	***
	L5-S1	882.1 ± 320.4	1080.8 ± 302.6	699.8 ± 209.2	***	482.8 ± 153.7	551.5 ± 139.5	419.7 ± 139.0	***
	L3-L4	790.0 ± 337.9	1038.5 ± 291.5	561.8 ± 179.8	***	366.4 ± 113.4	422.8 ± 98.6	314.7 ± 101.2	***
Right	L4-L5	1010.3 ± 364.7	1282.9 ± 294.5	760.0 ± 211.2	***	473.7 ± 144.3	549.9 ± 129.8	403.8 ± 120.1	***
	L5-S1	905.0 ± 337.3	1112.5 ± 338.2	720.3 ± 203.3	***	485.4 ± 149.0	558.0 ± 128.6	418.8 ± 135.3	***
	L3-L4	770.8 ± 304.6	1001.4 ± 243.7	559.1 ± 172.6	***	358.9 ± 106.6	412.7 ± 90.3	309.5 ± 96.5	***
Ave	L4-L5	1005.8 ± 333.9	1260.0 ± 266.5	772.4 ± 186.5	***	464.9 ± 140.5	546.1 ± 123.7	390.4 ± 111.0	***
	L5-S1	887.1 ± 319.5	1080.0 ± 319.2	710.0 ± 193.1	***	484.1 ± 144.6	554.7 ± 126.4	419.2 ± 129.6	***

The mean interobserver reliability was also good to excellent in measurement of the CSA of both PS and MF muscles in MRI. There were no significant differences between the three observers.

**Fig. 3:**
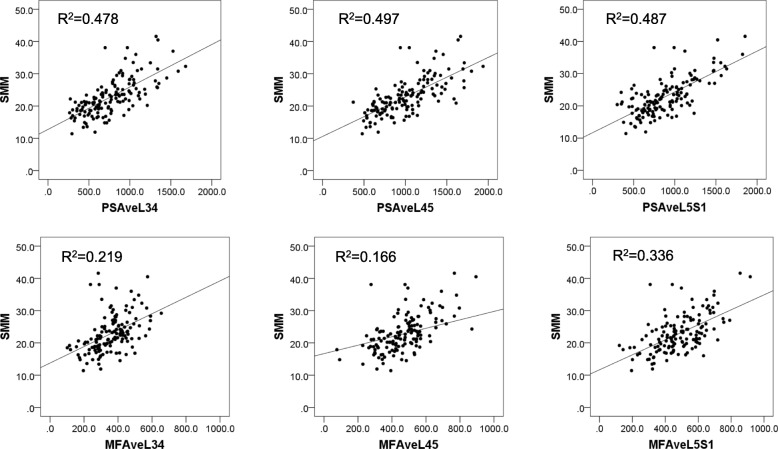
Correlation between SMM and PS AveCSA or MF AveCSA at the each level in 140 patients. SMM; skeletal muscle mass.

Analysis of the correlation between SMM and the AveCSA of each muscle was also performed for all lower lumbar levels. Correlations between SMM and PS AveCSA was found, in decreasing order of correlation coefficient, at L4-L5 (r = 0.739, *P* < 0.001), L3-L4 (r = 0.723, *P* < 0.001), and L5-S1 (r = 0.654, *P* < 0.001). Similarly, correlations between SMM and MF AveCSA were also found in L4-L5 (r = 0.582, *P* < 0.001), L5-S1 (r = 0.563, *P* < 0.001), and L3-L4 (r = 0.546, *P* < 0.001) (Table 4). These results demonstrated that PS AveCSA at the L4-L5 level was most positively correlated with SMM in patients with LSS and DS (Fig. 3). Correlation coefficient analysis showed weak correlation between SMM and PT (r = − 0.184, *P* < 0.05). Moreover, PS AveCSA (L4-L5) correlated with the PT (r = − 0.183, *P* < 0.05) and age (r = − 0.156, *P* < 0.05) (Table 5), while PT correlated with PS AveCSA (L4-L5) (r = − 0.183, *P* < 0.05) and whole body SMM (r = − 0.184, *P* < 0.05) but not with age. Analysis of the correlation between NRS of LBP and SMM did not show any correlation (SMM and NRS of LBP; r = − 0.014, *P =* 0.880). The mean score of NRS for 140 patients was 5.5 ± 2.8, and it did not show a statistically significant correlation with the PS AveCSA or MF AveCSA (Table 6).

**Table 4:** Correlation coefficient analysis of SMM and CSAs of PS and MF**.** PS; Psoas, MF; Multifidus, CSA; Cross-Sectional Area. ***p< 0.001 indicates significant differences between groups

	SMM	PS AveCSA (L3-L4)	PS AveCSA (L4-L5)	PS AveCSA (L5-S1)	MF AveCSA (L3-L4)	MF AveCSA (L4-L5)	MF AveCSA (L5-S1)
SMM	1.000	0.723***	0.739***	0.654***	0.546***	0.582***	0.563***
PS AveCSA (L3-L4)	0.723***	1.000	0.877***	0.646***	0.500***	0.585***	0.496***
PS AveCSA (L4-L5)	0.739***	0.877***	1.000	0.797***	0.509***	0.566***	0.506***
PS AveCSA (L5-S1)	0.654***	0.646***	0.797***	1.000	0.343***	0.393***	0.420***
MF AveCSA (L3-L4)	0.546***	0.500***	0.509***	0.343***	1.000	0.690***	0.538***
MF AveCSA (L4-L5)	0.582***	0.585***	0.566***	0.393***	0.690***	1.000	0.682***
MF AveCSA (L5-S1)	0.563***	0.496***	0.506***	0.420***	0.538***	0.682***	1.000

**Table 5:** Correlation coefficient analysis of spinal muscles, age and spinal alignment parameters**.** SMM; skeletal muscle mass, TK; thoracic kyphosis (T5–12), LL; lumbar lordosis (T12–S1), SS; sacral slope, PI; pelvic incidence, PT; pelvic tilt, SS; sacral slope, SVA; sagittal vertical axis, PS; Psoas. *p < 0.05, **< 0.01, ***< 0.001 indicates significant differences between groups

	SMM	Age	PS AveCSA (L4-L5)	TK	LL	PI	PT	SS	C7-SVA
SMM	1.000	−0.307***	–	−0.042	−0.067	−0.208*	−0.184*	−0.043	0.054
Age	−0.307***	1.000	−0.156*	0.063	0.006	0.056	0.055	0.051	0.171*
PS AveCSA (L4-L5)	0.739***	− 0.156*	1.000	0.014	−0.005	− 0.165*	− 0.183*	−0.004	0.035
TK	−0.042	0.063	0.014	1.000	0.490***	0.196*	−0.034	0.232*	−0.021
LL	−0.067	0.006	−0.005	0.490***	1.000	0.447***	−0.331***	0.780***	−0.516***
PI	−0.208*	0.056	−0.165*	0.196*	0.447***	1.000	0.447***	0.459***	−0.041
PT	−0.184*	0.055	−0.183*	−0.034	− 0.331***	0.447***	1.000	−0.498***	0.189*
SS	−0.043	0.051	−0.004	0.232*	0.780***	0.459***	−0.498***	1.000	−0.231*
C7-SVA	0.054	0.171*	0.035	−0.021	−0.516***	− 0.041	0.189*	− 0.231*	1.000

**Table 6:** Correlation coefficient analysis of NRS of LBP and CSAs of PS and MF**.** PS; Psoas, MF; Multifidus, CSA; Cross-Sectional Area. NRS; numeric rating scale, LBP; low back pain

	PS AveCSA (L3-L4)	PS AveCSA (L4-L5)	PS AveCSA (L5-S1)	MF AveCSA (L3-L4)	MF AveCSA (L4-L5)	MF AveCSA (L5-S1)
NRS	−0.026	0.004	0.080	−0.103	−0.198	− 0.103

## Discussion

There are several reports on the effects of aging on the morphology of the lumbar paraspinal muscles evaluated by MRI and the association of paraspinal muscle degeneration with LBP [7, 22]. We have also previously investigated whether SMM measured by BIA affects spinal alignment in patients with spinal degenerative disease and showed that SMM decreases with age [18]. To the best of our knowledge, this analysis is the largest investigation of the relationship between SMM and paraspinal muscle CSA in symptomatic LSS and/or DS patients. Our results show a high correlation between PS AveCSA (L4-L5) and whole body SMM and suggest a correlation with the spinal parameter PT.

Only a few studies have examined age-related changes in lumbar paraspinal muscle size, and these studies have reported inconsistent findings [1, 23, 24]. Takayama et al. examined the CSA of paraspinal muscles using T2-weighted MRI in 160 patients aged 10 to 88 years-old with an average lumbar lordosis of 20 degrees [23]. They demonstrated that the CSA of paraspinal muscles tended to decrease with age, which was also reported by Sasaki et al. [25]. On the other hand, Crawford et al. examined the volume of the erector spinae and multifidus muscles with 2-point Dixon 3 T MRI in 80 healthy volunteers aged 20 to 62, but the muscle volume did not depend on age [24]. Discrepancies between studies may be due to methodology, because differences in measurement techniques (CSA vs. volume), definition of paravertebral area, and research target population may influence the results.

In addition, males are known to have a larger CSA than females [23, 24]. In our analysis, as well as previous reports, there was a difference by gender, with larger PS and MF CSA in males than females. This result was similar for SMM measured by BIA. Chan et al. previously reported that male stenosis patients showed larger PS CSA than females, while elderly patients showed smaller PS CSA and more fat infiltration than younger stenosis patients [6]. Furthermore, in previous studies, the PS was known to exhibit the least fat infiltration, with Sasaki et al. reporting that the PS is highly unlikely to be affected by age-dependent degeneration [25]. However, our analysis suggests that SMM and PS AveCSA at the L4-L5 level are correlated, and that PS AveCSA at the L4-L5 has also a weak correlation with age.

To date, several studies reported the association between the size and fat content of the paraspinal muscles and LBP [3, 26, 27], but conflicting data has been obtained with regard to changes in PS CSA in cases of LBP. Arbanas et al. reported that LBP patients have significantly bigger PS CSA compared to control subjects [28], while Parkkola et al. described smaller PS CSA in patients with chronic LBP compared to healthy volunteers [27]. Unfortunately in this study, we were unable to determine whether PS CSA in patients with degenerative spine disease was smaller compared to healthy control subjects, as we focused only on patients with LSS and/or DS. However, we found that PS or MF CSA was highly correlated with whole body SMM, and that whole body SMM correlated with age. Regarding the relationship between LBP and paraspinal muscle, it is also known that there is a correlation between LBP and fatty infiltration of the paraspinal muscles [29]. In this study, we did not investigate fat infiltration, but focused on whole-body SMM and CSA of paraspinal muscles which showed no correlation between the CSA of paraspinal muscles and the intensity of LBP. These findings suggest that degeneration of paraspinal muscles and decrease in whole body SMM do not directly cause LBP. Or putting it into different terms, age-related degeneration of paraspinal muscles may not strongly correlate with LBP.

In large cohorts looking into spinal parameters in adult patients with degenerative lumbar disease, LL, PT, and PI were significantly smaller in males than in females, SVA, TK, and PT increased with age, and LL was reported to decrease with age [30]. In this study, we examined the relationship between spinal parameters, the paraspinal muscles, and SMM, because although it is well known that the spinal column and ligaments are important for maintaining spinal alignment, the relationship between sagittal alignment and paraspinal muscle CSA has not been sufficiently examined. MF and PS plays a role in the segmental stability of the lumbar spine [31, 32]. The MF and PS are also important for the motor control of the pelvis because they are directly attached to the pelvis. In this study, we found that the correlation between SMM and PS Ave CSA was higher than that between SMM and MF Ave CSA. The results suggests that PS may be related to anterior and posterior pelvic tilting than MF in patients with lumbar degenerative diseases. We also found that reduction of whole body SMM and decrease in PS CSA was correlated with PT. In other words, we believe that the inclination of the pelvis due to the reduction of SMM and especially the paraspinal muscles might be one factor that precipitates the onset of LBP. There are several limitations in our study. Study design prevents a strong conclusion about the role of MF and PS in spinal alignment or LBP. Longitudinal analysis is needed to determine whether a loss of muscle mass results in a spinal deformity or a spinal deformity results in a loss of muscle mass. Furthermore, the small number of participants and the lack of a control group limits the analytic power of the results. In this study, only patients with degenerative spinal disease were compared and not healthy volunteers. In order to confirm the results of our research, further studies recruiting more participants and a control group will be necessary. Furthermore, in this analysis, we did not analyze for adult patients with spinal deformities (kyphosis and scoliosis) such as Parkinson’s disease. The loss of muscle mass can cause disruption of the balance between extensors and flexors muscles of the spine. This imbalance along with all alterations take place in different parts of the spine, might have a consequence of spinal deformity. Finally, in this study, we focused on CSA of the paraspinal muscles and did not evaluate paraspinal muscles density because previous reports concerning fat infiltration in LBP patients were inconsistent. However, additional studies will be necessary to improve our understanding of the association and causal relationships between changes in the paraspinal muscle CSA or density and spinal alignment as well as LBP.

## Conclusions

Whole body SMM and paraspinal muscle CSA in 140 LSS and/or DS patients were evaluated. The whole body SMM showed strong correlation with PS AvCSA (L4-L5). In addition, whole body SMM as well as PS AvCSA (L4-L5) correlated with the spinal parameter PT. A significant correlation between PS AvCSA and PT suggests a causal relationship between muscle function and global spinal alignment. Inferring from randomized controlled trials conducted so far, intensive exercise programs can improve muscular strength, density and increase CSA of paraspinal muscles in LBP patients [33, 34], but this cannot be confirmed in this retrospective study. In the future, it will be necessary to analyze the influence of these paraspinal muscle strength and LBP with a prospective multicenter study.

## Data Availability

Data available upon request from corresponding author.

## Abbreviations

BIA: Bioimpedance analysis
BMI: Body mass index
CSA: Cross-sectional area
DS: Degenerative spondylolisthesis
LBP: Low back pain
LSS: Lumbar spinal stenosis
MF: Multifidus
NRS: Numeric rating scale
PS: Psoas major
QOL: Quality of life
SMM: Skeletal muscle mass
